# Twelve-Month Health-Related Quality of Life Recovery Following COVID-19 Hospitalization: A Prospective Cohort Study from Lithuania

**DOI:** 10.3390/medicina61091657

**Published:** 2025-09-11

**Authors:** Edita Strumiliene, Laura Malinauskiene, Birute Zablockiene, Ligita Jancoriene

**Affiliations:** 1 Clinic of Infectious Diseases and Dermatovenerology, Institute of Clinical Medicine, Faculty of Medicine, Vilnius University, LT-01513 Vilnius, Lithuanialigita.jancoriene@santa.lt (L.J.); 2 Clinic of Chest diseases, Immunology and Allergology, Institute of Clinical Medicine, Faculty of Medicine, Vilnius University, LT-01513 Vilnius, Lithuania; laura.malinauskiene@santa.lt

**Keywords:** COVID-19, quality of life, surveys and questionnaires, prospective studies, rehabilitation

## Abstract

*Background and Objectives*: As SARS-CoV-2 transitions toward endemic circulation, understanding long-term health impacts on quality of life (HRQoL) is critical for healthcare planning. While most longitudinal HRQoL studies originate from Western settings, data from Central and Eastern Europe remain scarce. This study aimed to track HRQoL changes over 12 months and explore the sociodemographic and clinical predictors of recovery in Lithuania. *Materials and Methods*: We conducted a prospective cohort study of 93 adults hospitalized with severe or critical COVID-19 at Vilnius University Hospital from October 2021 to October 2022. HRQoL was assessed at 3, 6, and 12 months post-discharge using the Short Form-36 Health Survey (SF-36). Longitudinal changes were analyzed using non-parametric tests, with minimal clinically important differences (MCIDs) applied. Multivariable regression identified predictors of 12-month outcomes. *Results*: Ninety-three participants (mean age 58.2 years; 53.8% female; 60.2% with critical disease; 95.7% unvaccinated) completed all follow-up visits. Seven of eight SF-36 domains showed clinically meaningful improvement over 12 months, most notably Bodily Pain (+18.8 points, r = 0.41), General Health (+14.6, r = 0.42), and Social Functioning (+10.4, r = 0.38). Role-Emotional improved minimally (+3.6, r = 0.16). Better Physical Functioning at 12 months was independently associated with male sex, employment, and fewer comorbidities. HRQoL scores remained below age-matched population norms. Only 12.9% accessed structured (Stage II) rehabilitation. *Conclusions*: This is the first comprehensive 12-month SF-36–based HRQoL assessment among hospitalized COVID-19 survivors in Central and Eastern Europe. Most domains improved significantly; however, emotional and social deficits persisted. Interpretation is limited by the single-center design, absence of pre-COVID baseline data, and use of a generic HRQoL measure. Low rehabilitation uptake underscores gaps in post-COVID care, highlighting the need for integrated, equity-focused recovery programs.

## 1. Introduction

The COVID-19 pandemic has left many survivors with lasting physical, psychological, and social impairments, collectively referred to as post-acute sequelae of SARS-CoV-2 infection (PASC) or long COVID [1,2,3,4,5,6]. Health-related quality of life (HRQoL) is a key recovery metric, reflecting patient-perceived well-being across multiple dimensions. The 36-Item Short-Form Health Survey (SF-36) is a widely validated instrument for assessing such multidimensional outcomes [7,8].

Although global studies have documented HRQoL impairments up to 12 months after COVID-19, most are from Western Europe, North America, or Asia, often involving outpatients or general populations [9,10,11]. Data from Central and Eastern Europe, particularly in hospitalized cohorts, are scarce. Lithuania—which is among the European countries with the highest per capita COVID-19 mortality [12]—lacks comprehensive SF-36–based evaluations of post-hospitalization recovery. Existing regional studies have largely relied on symptom checklists, qualitative assessments, or generic tools such as the EQ-5D or Hospital Anxiety and Depression Scale (HADS) [10,11], providing limited insight into domain-specific HRQoL recovery.

Recent European cohorts underscore the persistence of post-COVID deficits. In Italy, SF-36-based follow-up of hospitalized patients demonstrated that physical quality of life gradually improved, whereas mental health remained persistently impaired for up to three years after discharge [13]. In the Netherlands, the CO-FLOW multicenter study tracked survivors for 24 months, reporting incomplete recovery and enduring functional limitations [14]. Similarly, German longitudinal analyses using the SF-36 found improvements in the Role-Physical and social domains, but sustained deficits in vitality and mental health [15]. These studies highlight heterogeneous recovery trajectories across Europe and the importance of national context in shaping outcomes.

Rehabilitation is increasingly well recognized as integral to post-COVID management, yet referral pathways, timing, and optimal resource allocation remain poorly defined in resource-limited healthcare systems. Identifying the patient subgroups most likely to benefit could improve recovery outcomes and efficiency [16].

This prospective cohort study aimed to (1) track changes in all eight SF-36 domains at 3, 6, and 12 months after hospital discharge; (2) identify demographic and clinical predictors of recovery using minimal clinically important differences (MCIDs); (3) compare 12-month outcomes with age-matched population norms; and (4) assess access to rehabilitation services. The findings aim to inform targeted, sustainable post-COVID care strategies in similar healthcare contexts.

## 2. Materials and Methods

### 2.1. Study Design and Setting

This prospective observational cohort study was conducted at Vilnius University Hospital Santaros Klinikos, the largest tertiary care and national referral center in Lithuania for severe and critical COVID-19 cases.

### 2.2. Participants

Eligible participants were adults (≥18 years) hospitalized with laboratory-confirmed SARS-CoV-2 infection (verified by RT-PCR from a nasopharyngeal swab) and radiologically confirmed lung injury between October 2021 and October 2022.

Exclusion criteria included cognitive impairment precluding valid questionnaire completion, terminal illness with a life expectancy of <12 months, and inability or unwillingness to provide informed consent.

Of 130 eligible patients, 122 (93.8%) consented to participate. Among these, 93 patients (71.5% of those eligible; 76.2% of those enrolled) completed all three follow-up visits and were included in the final analysis. Data were collected during face-to-face visits at the hospital by trained investigators. Participants were contacted by telephone to schedule follow-up visits, which included a structured clinical interview, symptom assessment, and venous blood sampling.

The primary reasons for loss to follow-up were logistical or personal constraints. Baseline characteristics did not significantly differ between those who completed the study and those who did not.

### 2.3. COVID-19 Severity Classification

COVID-19 severity was classified using a modified World Health Organization Clinical Progression Scale [17], which was adapted to reflect national triage thresholds:

- Severe disease: Bilateral pneumonia with >50% lung involvement and either respiratory rate ≥ 30 breaths/min, oxygen saturation ≤ 93% on room air, or oxygen requirement ≤ 10 L/min, without ICU admission.
- Critical disease: Respiratory failure requiring high-flow oxygen (>10 L/min), mechanical ventilation, vasopressors, or intensive care unit admission due to hemodynamic instability or multi-organ dysfunction.

### 2.4. HRQoL Assessment

Health-related quality of life was assessed using the validated Lithuanian version of the SF-36 [18]. This instrument evaluates eight domains: Physical Functioning (PF), Role-Physical (RP), Bodily Pain (BP), General Health (GH), Vitality (VT), Social Functioning (SF), Role-Emotional (RE), and Mental Health (MH). Scores in each domain ranged from 0 to 100, with higher values reflecting better health status. Questionnaires were administered via structured clinical interviews at 3, 6, and 12 months post-discharge.

### 2.5. Statistical Analysis

A post hoc power analysis confirmed that with a sample size of 93, the study had >80% power to detect medium effect sizes (r ≥ 0.30) at α = 0.05. Continuous variables are presented as means ± standard deviations, and categorical variables as frequencies and percentages. Data normality was assessed using Shapiro–Wilk tests. Because all SF-36 domain scores were non-normally distributed (all *p* < 0.05), longitudinal changes were analyzed using Friedman tests with post hoc Wilcoxon signed-rank tests. Bonferroni correction was applied (adjusted α = 0.017 for three pairwise comparisons; adjusted α = 0.006 for eight domain comparisons). Effect sizes were calculated using rank-biserial correlation (r), interpreted as small (≥0.10), moderate (≥0.30), or large (≥0.50) [19], and 95% confidence intervals were derived using bias-corrected accelerated bootstrapping with 1000 resamples.

Clinical significance was evaluated using established minimal clinically important differences (MCIDs): Physical Functioning (≥10 points), Role-Physical (≥25 points), Bodily Pain (≥10 points), General Health (≥5 points), Vitality (≥10 points), Social Functioning (≥12.5 points), Role-Emotional (≥25 points), and Mental Health (≥5 points) [20,21]. Between-group comparisons were performed with Mann–Whitney U tests (two groups) and Kruskal–Wallis tests (≥3 groups), with Bonferroni correction applied.

Independent predictors of 12-month outcomes were identified using multiple linear regression. Model assumptions were verified through residual diagnostics, including Shapiro–Wilk tests, Q–Q plots, and assessments of homoscedasticity and independence. When parametric assumptions were violated, quantile regression analyses were conducted to confirm robustness.

Twelve-month SF-36 scores were compared with age-matched Lithuanian population norms (50–65 years) using Wilcoxon signed-rank tests [22]. All analyses were performed with Python 3.9 and R version 4.3.0. Statistical significance was defined as *p* < 0.05. Non-parametric methods were chosen for longitudinal comparisons due to non-normal distributions, while regression modeling was considered appropriate because its assumptions pertain to residuals, not raw data; diagnostics confirmed model validity.

### 2.6. Comparison with Population Norms

Twelve-month SF-36 scores were compared to age-matched Lithuanian adults aged 50–65, derived from European Quality of Life Survey data [22]. For consistency with our longitudinal analysis approach, we primarily used Wilcoxon signed-rank tests to compare 12-month scores with population norms. One-sample *t*-tests were used as sensitivity analyses where 12-month score distributions approached normality (Shapiro–Wilk *p* > 0.05). Both parametric and non-parametric results were consistent in direction and significance.

### 2.7. Rehabilitation Classification

Rehabilitation participation was classified according to national healthcare categories:

- No rehabilitation: Patients discharged without structured rehabilitation services.
- Stage I rehabilitation: Inpatient rehabilitation during hospitalization (physiotherapy, occupational therapy, or respiratory therapy).
- Stage II rehabilitation: Post-discharge structured multidisciplinary programs (outpatient or sanatorium-based) lasting 2–4 weeks.
- Rehabilitation referrals during the study period were not standardized but were determined by individual clinicians’ judgment and local resource availability, which may have contributed to the low uptake of structured (Stage II) rehabilitation.

### 2.8. Ethics

This study was approved by the Vilnius Regional Biomedical Research Ethics Committee (Protocol No. 2020/6-1233-718; approved 22 June 2020) and conducted according to the Declaration of Helsinki. All participants provided written informed consent.

## 3. Results

### 3.1. Cohort Characteristics

The final cohort included 93 patients (mean age 58.2 ± 10.2 years; 53.8% female). Critical COVID-19 occurred in 56 (60.2%) of patients. Only four patients (4.3%) were vaccinated at the time of infection. Comorbidities were frequent: 34.4% had none, 35.5% had 1, and 30.1% had ≥2. The most common comorbidities were hypertension (48.4%), diabetes mellitus (24.7%), and cardiovascular disease (20.4%). The comprehensive baseline characteristics of the study cohort are shown in Table 1.

### 3.2. Changes in HRQoL over Time

**Figure 1:**
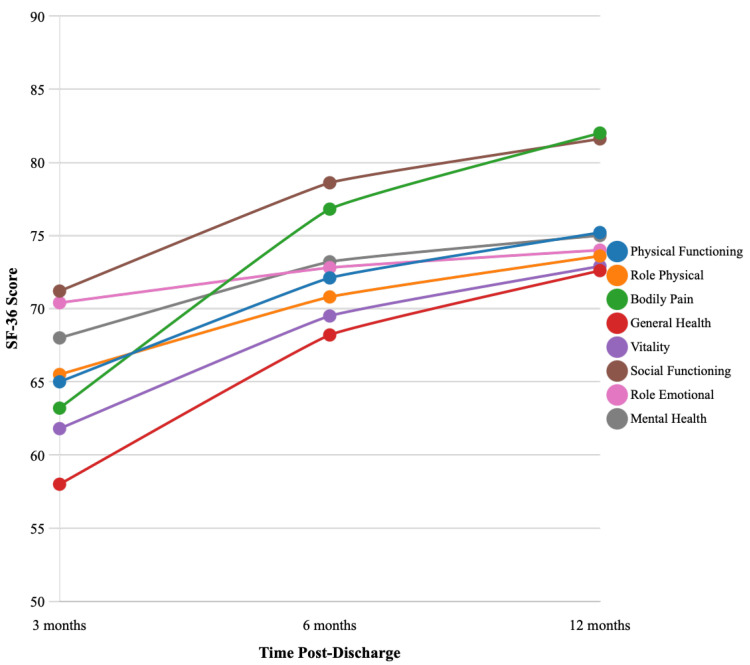
SF-36 Recovery Trajectories Over 12 Months. Line graph showing mean SF-36 domain scores at 3, 6, and 12 months post-discharge. Error bars represent 95% confidence intervals. Horizontal dashed lines indicate minimal clinically important differences (MCIDs) for each domain to enable visual assessment of both statistical and clinical significance. Seven out of eight domains achieved clinically meaningful improvements (effect size r ≥ 0.30), with Role-Emotional showing minimal change (r = 0.16).

All eight SF-36 domains improved significantly from 3 to 12 months (Friedman test, *p* < 0.001). Seven of these domains demonstrated both statistically significant and clinically meaningful changes (Table 2, Figure 1).

Domains with largest improvements:

- Bodily Pain: improved by +18.8 points (effect size r = 0.41, 95% CI: 0.25–0.57), surpassing the MCID (≥10 points).
- General Health: improved by +14.6 points (r = 0.42, 95% CI: 0.26–0.58), well above the MCID (≥5 points).
- Social Functioning: improved by +10.4 points (r = 0.38, 95% CI: 0.22–0.54), approaching the MCID (≥12.5 points).

Domains with moderate improvements:

- Physical Functioning: +10.2 points (r = 0.37, 95% CI: 0.21–0.53), meeting the MCID (≥10 points).
- Role Physical: +8.1 points (r = 0.34, 95% CI: 0.18–0.50), below the MCID (≥25 points).
- Mental Health: +7.0 points (r = 0.32, 95% CI: 0.16–0.48), exceeding the MCID (≥5 points).
- Vitality: +11.1 points (r = 0.30, 95% CI: 0.14–0.46), meeting the MCID (≥10 points).

Domain with minimal improvement:

- Role-Emotional: +3.6 points (r = 0.16, 95% CI: 0.02–0.30), well below the MCID (≥25 points) indicating limited recovery in emotional role functioning.

### 3.3. Comparison with Age-Matched Population Norms

**Figure 2:**
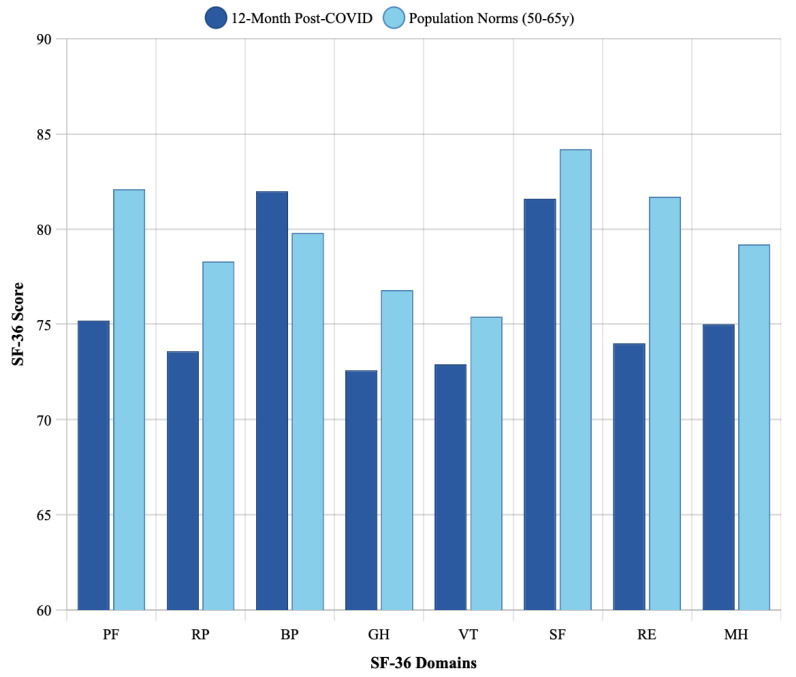
Comparison of 12-month post-COVID SF-36 scores with age-matched population norms. Bar chart comparing mean 12-month SF-36 domain scores (dark bars) with Lithuanian population norms for ages 50–65 years (light bars). Patients approached but did not reach population norms in most domains, with the largest gaps recorded for Physical Functioning (6.9 points) and Role-Emotional (7.7 points).

At 12 months, patients had not fully returned to pre-pandemic age-matched population norms (Table 3, Figure 2). The largest gaps were observed in Physical Functioning (75.2 vs. 82.1, gap = 6.9 points; *p* = 0.003), Role-Physical (73.6 vs. 78.3, gap = 4.7 points), and General Health (72.6 vs. 76.8, gap = 4.2 points; *p* = 0.045). Mental Health scores nearly reached population norms (75.0 vs. 79.2, gap = 4.2 points), but remained slightly lower.

### 3.4. Predictors of Recovery at 12 Months

Multivariate linear regression models identified key predictors for better outcomes in key HRQoL domains: (Table 4).

Physical Functioning (R^2^ = 0.42, *p* < 0.001):

- Male sex: β = 14.60, 95% CI: 6.50–22.70, *p* < 0.001;
- Fewer comorbidities: β = −10.76, 95% CI: −16.20 to −5.32, *p* < 0.001;
- Employment: β = 8.45, 95% CI: 2.10–14.80, *p* = 0.010.

Mental Health (R^2^ = 0.31, *p* = 0.002):

- Male sex: β = 10.89, 95% CI: 3.62–18.16, *p* = 0.004;
- Employment: β = 6.73, 95% CI: 0.95–12.51, *p* = 0.023.

General Health (R^2^ = 0.28, *p* = 0.006):

- More comorbidity: β = −7.46, 95% CI: −13.82 to −1.10, *p* = 0.021;
- Older age: β = −0.42, 95% CI: −0.78 to −0.06, *p* = 0.024.

### 3.5. Disparities in Recovery by Demographic Characteristics

**Figure 3:**
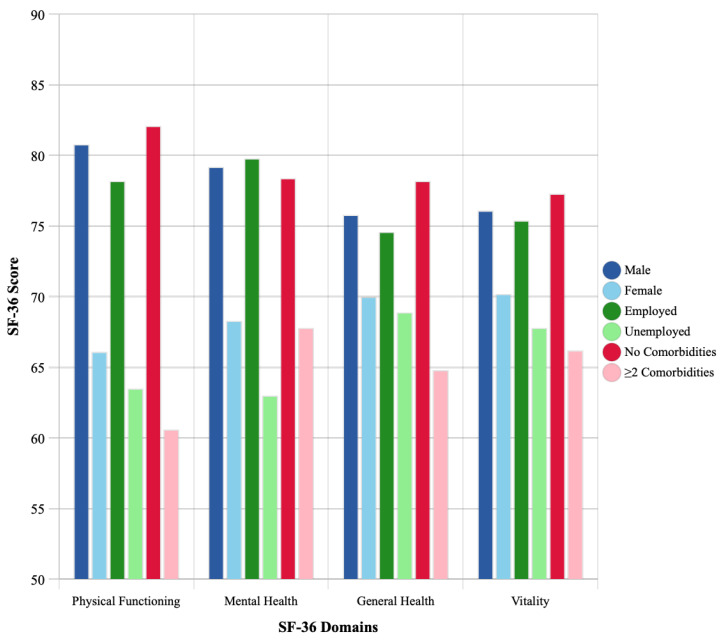
Sociodemographic disparities in HRQoL. Multi-panel figure showing 12-month SF-36 domain scores stratified by gender (male vs. female), employment status (employed vs. unemployed), and comorbidity burden (none, one, and ≥two). Box plots display median, interquartile ranges, and outliers. Pronounced disparities were observed across all sociodemographic factors, with males, employed individuals, and those with fewer comorbidities achieving superior outcomes.

Significant 12-month outcome disparities were evident across sex, employment status, and comorbidity burden (Table 5, Figure 3):

Gender differences:

- Physical Functioning: males scored 80.8 ± 16.2 vs. females 66.1 ± 20.1 (*p* < 0.001);
- Mental Health: males 79.2 ± 15.4 vs. females 68.3 ± 18.7 (*p* = 0.002).

Employment disparities:

- Physical Functioning: employed 78.2 ± 17.1 vs. unemployed 63.5 ± 21.3 (*p* = 0.001);
- Mental Health: employed 79.8 ± 16.2 vs. unemployed 63.0 ± 19.4 (*p* < 0.001).

Comorbidities:

- Individuals with ≥2 comorbidities had significantly lower scores across most domains. For example, Physical Functioning obtained a score of 82.1± 14.6 with no comorbidities vs. 60.6± 22.8 with two or more (*p* < 0.001).

### 3.6. Healthcare Utilization and Access to Rehabilitation

During 12-month follow-up, a substantial proportion of patients continued to require medical care: 47.3% (*n* = 44) had pulmonology consultations, 22.6% (*n* = 21) had cardiology follow-up, 6.5% (*n* = 6) required home oxygen, and 5.4% (*n* = 5) experienced pulmonary embolism.

Despite patients’ ongoing healthcare needs, rehabilitation utilization remained low: 58.1% (*n* = 54) received no rehabilitation; 29.0% (*n* = 27) underwent inpatient rehabilitation (Stage I); and only 12.9% (*n* = 12) accessed structured post-discharge rehabilitation (Stage II).

Patients receiving Stage II rehabilitation demonstrated higher 12-month scores for Physical Functioning (78.2 ± 15.6 vs. 72.8 ± 19.4, *p* = 0.32) and Mental Health (73.6 ± 15.4 vs. 71.2 ± 17.8, *p* = 0.65), though differences lacked statistical significance due to the small sample size.

## 4. Discussion

### 4.1. Key Findings

This is the first study from Lithuania, a Central and Eastern European country, to provide a comprehensive, domain-specific assessment of 12-month HRQoL recovery in hospitalized COVID-19 survivors using the SF-36 instrument. In a predominantly unvaccinated Lithuanian cohort, we found substantial and clinically meaningful improvements in seven out of eight domains, underscoring the potential for significant recovery even after severe or critical illness.

The largest gains were observed in Bodily Pain (+18.8 points, r = 0.41) and General Health (+14.6 points, r = 0.42), accompanied by a notable improvement in Social Functioning (+10.4 points, r = 0.38). These changes indicate recovery that extends beyond physical capacity to include pain relief, enhanced health perception, and reintegration into social roles.

However, Role-Emotional showed minimal change (+3.6 points, r = 0.16), highlighting persistent limitations in emotionally demanding activities and the need for targeted psychological support within post-COVID care pathways. Persistent deficits in Role-Emotional functioning are consistent with recent European studies [23,24], which describe ongoing psychological and social sequelae despite improvements in physical domains. Limited access to specialized mental health services, particularly in resource-limited post-pandemic settings, may further contribute to incomplete recovery in this domain. Importantly, despite improvements, HRQoL scores remained below age-matched population norms across multiple domains, reflecting incomplete recovery one year after discharge [24].

### 4.2. Clinical Relevance of Changes

The use of established MCIDs allowed for interpretation beyond statistical significance. Improvements in bodily pain and general health exceeded MCID thresholds, suggesting meaningful relief in symptoms and perceived well-being. Physical Functioning, Mental Health, and Vitality improvements met or exceeded their MCIDs, supporting functional and psychological recovery.

In contrast, Role-Physical and Role-Emotional improvements fell below their respective MCIDs. This indicates that although patients may experience improved daily functioning, their ability to resume occupational or emotionally demanding roles remains limited, highlighting a key area for targeted post-COVID interventions.

### 4.3. Context in the Global Literature

This study is one of the first to provide comprehensive, domain-specific HRQoL outcomes using the SF-36 instrument in a Central and Eastern European context. The finding that seven domains achieved clinically meaningful recovery contrasts with some Western studies reporting more persistent impairments [25,26]. However, our predominantly unvaccinated cohort (95.7%) may explain these differences, as vaccination status significantly influences both acute illness severity and long-term recovery patterns [23,27].

The marked Bodily Pain improvement represents one of the largest pain recovery effects documented in post-COVID literature, addressing a critical gap, as pain remains among the most common but understudied long-COVID symptoms [24,28]. Social Functioning recovery (r = 0.38) is particularly noteworthy, due to the existence of profound pandemic-related social isolation, suggesting that post-acute care interventions should incorporate social reintegration components.

### 4.4. Sociodemographic Disparities and Health Equity

One of the most striking findings is the disparity in recovery across sociodemographic lines. Male patients and those who were employed consistently demonstrated better physical and mental health outcomes exceeding established MCIDs [20,21]. These findings align with emerging research showing that socioeconomic factors are consistently associated with poorer post-COVID recovery [29,30].

Gender disparities were pronounced, with males achieving superior outcomes across domains. This pattern, which has been observed internationally, may reflect biological, psychological, and social factors influencing recovery [31,32]. The clear dose–response relationship between comorbidity burden and outcomes underscores vulnerability among patients with multiple chronic conditions, emphasizing the need for integrated chronic disease management in post-COVID care [33,34].

The inverse relationship between comorbidity burden and HRQoL further highlights vulnerability among individuals with multiple chronic conditions. That such disparities persisted in Lithuania’s universal healthcare system suggests that structural or social factors—such as income, caregiving roles, or social support—may play a larger role in recovery than access alone. It should be noted that our rehabilitation utilization analysis is limited by small sample sizes, particularly for Stage II rehabilitation (*n* = 12). While we observed numerically higher scores among patients receiving structured rehabilitation, definitive conclusions about effectiveness require larger studies with adequate power for subgroup comparisons.

### 4.5. Healthcare System and Rehabilitation Implications

This study revealed a disconnect between patient needs and service utilization. Nearly half of participants required specialist follow-up for respiratory or cardiac concerns, yet only 12.9% accessed structured Stage II rehabilitation. Although Lithuania expanded rehabilitation infrastructure during the pandemic, utilization during the study period remained critically low. This gap may reflect unclear eligibility criteria, insufficient patient awareness, logistical barriers such as travel and scheduling, and suboptimal healthcare provider referral patterns [35,36].

The disparity between high medical needs and low rehabilitation uptake likely contributed to incomplete recovery and may increase long-term healthcare costs. More efficient integration of medical and rehabilitative care—particularly through clearer referral pathways and improved accessibility—could enhance outcomes and reduce the strain on healthcare systems, especially in resource-constrained settings.

## 5. Clinical Implications and Recommendations

Based on our findings, several clinical priorities emerge:

**Risk Stratification:** Rehabilitation services should prioritize high-risk individuals—those who are unemployed, female, or with multiple comorbidities. **Integrated Care Pathways:** Post-COVID management should bridge medical and rehabilitative services, especially for those with persistent symptoms. **Pain Management:** Programs should include structured approaches to pain management and psychological support, particularly for emotional role recovery. **Social Reintegration:** Interventions that address social functioning may accelerate overall recovery and improve mental well-being.

## 6. Strengths and Limitations

Several methodological and contextual factors temper the generalizability and interpretation of these findings. First, the single-center design and absence of pre-COVID baseline data limit interpretation. Moreover, as the majority of our cohort was unvaccinated, the findings may not be fully comparable to contemporary cohorts of vaccinated patients, in whom disease severity and recovery trajectories may differ. Second, the use of SF-36—while validated and multidimensional—may underrepresent domains particularly relevant to post-COVID sequelae, such as cognitive impairment, severe fatigue, and organ-specific limitations. This limitation should be considered when comparing results with studies employing more targeted instruments.

The interpretation of clinically meaningful change was based on MCID thresholds derived from general medical populations. Whether these thresholds fully capture patient-perceived recovery in post-viral syndromes remains uncertain. Moreover, while improvements were evident relative to 3-month post-discharge status, the absence of pre-COVID HRQoL data precludes conclusions about return to personal baseline functioning. Comparisons with age-matched population norms, though informative, do not account for the higher baseline comorbidity burden of the study cohort.

Observed disparities by sex, employment status, and comorbidity burden were consistent with international findings, yet causality cannot be inferred. Potential confounders—such as pre-COVID physical fitness, caregiving responsibilities, or differential healthcare-seeking behaviors—were not measured. Similarly, while low Stage II rehabilitation uptake points to potential systemic barriers, patient preference, symptom severity, and logistical constraints may also explain limited utilization. Given the small sample in the Stage II subgroup, observed outcome differences should be interpreted with caution.

Despite these caveats, the data suggest that recovery from pain, perceived health, and social engagement can be achieved within 12 months for many hospitalized COVID-19 survivors. However, persistent emotional role limitations and incomplete return to normative functioning point to the need for integrated care pathways that address both physical and psychosocial dimensions of recovery.

The key strengths of this study include its prospective design with systematic recruitment, the high follow-up completion rate (100%) among enrolled participants, the validated HRQoL instrument with MCID application, the comprehensive 12-month follow-up, the population norm comparisons, and data from an understudied Central and Eastern European population.

## 7. Future Research Directions

Future investigations should focus on developing integrated care pathways coordinating ongoing medical interventions with rehabilitation services, evaluating the cost-effectiveness of comprehensive post-COVID care models, and investigating optimal protocols for identifying patients who will benefit most from structured rehabilitation. Cross-national studies comparing post-viral recovery patterns across Central and Eastern and Western European healthcare systems would provide valuable insights into how healthcare infrastructure influences long-term outcomes.

## 8. Conclusions

This is the first comprehensive study in Lithuania, and among the first in Central and Eastern Europe, to assess 12-month HRQoL recovery using SF-36, revealing significant, clinically meaningful gains across most domains over a 12-month period. Significant and clinically meaningful improvements were observed in most SF-36 domains, particularly General Health, Bodily Pain, and Social Functioning. However, patients did not fully regain population-norm levels, and emotional role limitations remained persistent.

Importantly, disparities in recovery outcomes were evident across gender, employment status, and comorbidity burden, with vulnerable subgroups showing slower improvement. These findings highlight the need for more targeted, equity-focused post-COVID care strategies that address both physical and psychosocial recovery.

The low utilization of rehabilitation services despite ongoing healthcare needs underscores systemic gaps in care delivery during the pandemic period. Sustainable care models should integrate medical management, psychological support, and rehabilitation, especially for vulnerable populations.

Interpretation should account for the study’s single-center design, predominantly unvaccinated population, absence of pre-COVID baseline data, and reliance on SF-36 as a generalist measure. The results underscore that improvement does not necessarily equate to full recovery and that persistent deficits—particularly in emotional and role functioning—require sustained attention.

By capturing recovery patterns across eight HRQoL domains, this study offers valuable insights for planning long-term post-viral care strategies—not only for COVID-19 but also for future pandemics affecting similar populations and healthcare systems.

## Figures and Tables

**Table 1:** Baseline characteristics of study participants (*n*= 93).

Characteristic	*n* (%) or Mean ± SD
**Demographics**	
Age (years)	58.2 ± 10.2
Female sex	50 (53.8)
Employed	62 (66.7)
**COVID-19 Severity**	
Severe	37 (39.8)
Critical	56 (60.2)
**Vaccination Status**	
Unvaccinated	89 (95.7)
Vaccinated	4 (4.3)
**Comorbidities**	
None	32 (34.4)
One	33 (35.5)
Two or more	28 (30.1)
Hypertension	45 (48.4)
Diabetes mellitus	23 (24.7)
Cardiovascular disease	19 (20.4)
**Rehabilitation Status**	
No rehabilitation	54 (58.1)
Stage I only	27 (29.0)
Stage II	12 (12.9)

**Table 2:** SF-36 domain scores over time and clinical significance (*N* = 93).

Domain	3 Months Mean ± SD	6 Months Mean ± SD	12 Months Mean ± SD	Change (3–12 Months)	Effect Size r (95% CI)	MCID Threshold	Clinical Significance
Physical Functioning	65.0 ± 22.1	72.1 ± 19.8	75.2 ± 18.5	+10.2	0.37 (0.21–0.53)	≥10	Yes
Role Physical	65.5 ± 28.3	70.8 ± 26.1	73.6 ± 25.4	+8.1	0.34 (0.18–0.50)	≥25	No
Bodily Pain	63.2 ± 24.6	76.8 ± 22.1	82.0 ± 20.3	+18.8	0.41 (0.25–0.57)	≥10	Yes
General Health	58.0 ± 18.9	68.2 ± 17.6	72.6 ± 16.8	+14.6	0.42 (0.26–0.58)	≥5	Yes
Vitality	61.8 ± 20.4	69.5 ± 18.7	72.9 ± 17.9	+11.1	0.30 (0.14–0.46)	≥10	Yes
Social Functioning	71.2 ± 25.8	78.6 ± 22.4	81.6 ± 21.1	+10.4	0.38 (0.22–0.54)	≥12.5	Approaching
Role Emotional	70.4 ± 29.2	72.8 ± 27.6	74.0 ± 26.8	+3.6	0.16 (0.02–0.30)	≥25	No
Mental Health	68.0 ± 18.5	73.2 ± 17.1	75.0 ± 16.9	+7.0	0.32 (0.16–0.48)	≥5	Yes

All domains showed significant improvement over time (Friedman test, *p* < 0.001 after Bonferroni correction). MCID = minimal clinically important difference.

**Table 3:** Twelve-month SF-36 scores compared to population norms (Ages 50–65).

Domain	Post-COVID 12-Month Mean ± SD	Population Norm (50–65 Years)	Gap	*p*-Value *
Physical Functioning	75.2 ± 18.5	82.1	6.9	0.003
Role-Physical	73.6 ± 25.4	78.3	4.7	0.12
Bodily Pain	82.0 ± 20.3	79.8	−2.2	0.38
General Health	72.6 ± 16.8	76.8	4.2	0.045
Vitality	72.9 ± 17.9	75.4	2.5	0.26
Social Functioning	81.6 ± 21.1	84.2	2.6	0.33
Role-Emotional	74.0 ± 26.8	81.7	7.7	0.021
Mental Health	75.0 ± 16.9	79.2	4.2	0.052

* One-sample *t*-test comparing post-COVID scores to population norms.

**Table 4:** Multivariable linear regression models predicting 12-month SF-36 outcomes.

Variable	Physical Functioning β (95% CI)	Mental Health β (95% CI)	General Health β (95% CI)
**Male gender**	**14.60 (6.50–22.70)** *	**10.89 (3.62–18.16)**	5.23 (−1.84–12.30)
**Age (years)**	−0.21 (−0.58–0.16)	−0.15 (−0.49–0.19)	**−0.42 (−0.78 to −0.06)**
**Employment**	**8.45 (2.10–14.80)**	**6.73 (0.95–12.51)**	3.82 (−1.95–9.59)
**Comorbidity burden**	**−10.76 (−16.20 to −5.32)** *	−5.14 (−10.08 to −0.20)	**−7.46 (−13.82 to −1.10)**
**Critical COVID-19**	−3.22 (−9.87–3.43)	−2.15 (−8.12–3.82)	−1.88 (−7.85–4.09)
**Stage II rehabilitation**	5.42 (−3.78–14.62)	2.31 (−6.11–10.73)	4.17 (−3.25–11.59)
**Model Statistics**			
R^2^	0.42	0.31	0.28
F-statistic	10.2 ***	6.4 **	5.5 **
RMSE	14.1	14.8	14.2

* *p* < 0.05, ** *p* < 0.01, *** *p* < 0.001. Bold indicates statistical significance.

**Table 5:** HRQoL scores by sociodemographic subgroups at 12 months.

Domain	Gender		*p*-Value	Employment		*p*-Value	Comorbidities			*p*-Value †
	Male (*n* = 43)	Female (*n* = 50)		Employed (*n* = 62)	Unemployed (*n* = 31)		None (*n* = 32)	One (*n* = 33)	≥Two (*n* = 28)	
**Physical Functioning**	80.8 ± 16.2	66.1 ± 20.1	<0.001	78.2 ± 17.1	63.5 ± 21.3	0.001	82.1 ± 14.6	74.8 ± 18.2	60.6 ± 22.8	<0.001
**Role Physical**	78.4 ± 23.8	69.6 ± 26.2	0.089	77.1 ± 24.2	65.8 ± 27.1	0.041	79.2 ± 22.1	73.6 ± 26.4	65.4 ± 28.9	0.098
**Bodily Pain**	85.2 ± 18.4	79.3 ± 21.6	0.142	83.8 ± 19.1	78.4 ± 22.8	0.223	87.1 ± 16.8	81.2 ± 20.8	76.8 ± 23.1	0.124
**General Health**	75.8 ± 15.9	70.0 ± 17.2	0.095	74.6 ± 16.1	68.9 ± 18.2	0.126	78.2 ± 14.5	72.1 ± 17.2	64.8 ± 18.4	0.008
**Vitality**	76.1 ± 16.8	70.2 ± 18.6	0.111	75.4 ± 17.2	67.8 ± 19.1	0.052	77.3 ± 15.9	72.8 ± 18.4	66.2 ± 19.8	0.045
**Social Functioning**	84.8 ± 19.2	78.9 ± 22.4	0.172	83.6 ± 20.1	77.4 ± 23.1	0.184	86.2 ± 17.8	81.4 ± 21.8	75.9 ± 24.2	0.156
**Role Emotional**	77.2 ± 25.1	71.3 ± 28.1	0.283	76.8 ± 25.9	68.4 ± 28.4	0.158	78.1 ± 24.2	74.2 ± 27.8	68.6 ± 29.1	0.345
**Mental Health**	79.2 ± 15.4	68.3 ± 18.7	0.002	79.8 ± 16.2	63.0 ± 19.4	<0.001	78.4 ± 15.8	75.2 ± 17.1	67.8 ± 18.9	0.032

Mann–Whitney U test for gender and employment; **†** Kruskal–Wallis test for comorbidities, with Bonferroni correction (adjusted α = 0.017) for multiple comparisons.

## Data Availability

The datasets used and analyzed during the current study are available from the corresponding author upon reasonable request, subject to appropriate ethical approvals and data sharing agreements in accordance with Lithuanian data protection regulations.

## References

[1] Nalbandian A., Sehgal K., Gupta A., Madhavan M.V., McGroder C., Stevens J.S., Cook J.R., Nordvig A.S., Shalev D., Sehrawat T.S. (2021). Post-acute COVID-19 syndrome. Nat. Med..

[2] Soriano J.B., Murthy S., Marshall J.C., Relan P., Diaz J.V. (2022). A clinical case definition of post-COVID-19 condition by a Delphi consensus. Lancet Infect. Dis..

[3] Huang C., Huang L., Wang Y., Li X., Ren L., Gu X., Kang L., Guo L., Liu M., Zhou X. (2021). 6-month consequences of COVID-19 in patients discharged from hospital: A cohort study. Lancet.

[4] Sudre C.H., Murray B., Varsavsky T., Graham M.S., Penfold R.S., Bowyer R.C., Pujol J.C., Klaser K., Antonelli M., Canas L.S. (2021). Attributes and predictors of long COVID. Nat. Med..

[5] Davis H.E., Assaf G.S., McCorkell L., Wei H., Low R.J., Re’EM Y., Redfield S., Austin J.P., Akrami A. (2021). Characterizing long COVID in an international cohort: 7 months of symptoms and their impact. EClinicalMedicine.

[6] Carfì A., Bernabei R., Landi F., Gemelli Against COVID-19 Post-Acute Care Study Group (2020). Persistent symptoms in patients after acute COVID-19. JAMA.

[7] Ware J.E., Sherbourne C.D. (1992). The MOS 36-item short-form health survey (SF-36). I. Conceptual framework and item selection. Med. Care.

[8] Gandek B., Ware J.E., Aaronson N.K., Apolone G., Bjorner J.B., Brazier J.E., Bullinger M., Kaasa S., Leplege A., Prieto L. (1998). Cross-validation of item selection and scoring for the SF-12 Health Survey in nine countries: Results from the IQOLA Project. J. Clin. Epidemiol..

[9] Malik P., Patel K., Pinto C., Jaiswal R., Tirupathi R., Pillai S., Patel U. (2022). Post-acute COVID-19 syndrome (PCS) and health-related quality of life (HRQoL)—A systematic review and meta-analysis. J. Med. Virol..

[10] Fernández-De-Las-Peñas C., Palacios-Ceña D., Gómez-Mayordomo V., Florencio L.L., Cuadrado M.L., Plaza-Manzano G., Navarro-Santana M. (2021). Prevalence of post-COVID-19 symptoms in hospitalized and non-hospitalized survivors: A systematic review and meta-analysis. Eur. J. Intern. Med..

[11] Harirchian M.H., Zahednasab H., Karampoor S. (2022). Long-term outcomes of hospitalized COVID-19 patients with and without neurological complications. J. Neurol. Sci..

[12] Statistics Lithuania (2023). COVID-19 Dashboard. https://osp.stat.gov.lt/covid-dashboards.

[13] Berentschot J.C., Bek L.M., Drost M., van den Berg-Emons R.J.G., Braunstahl G.J., Ribbers G.M., Aerts J.G.J.V., Hellemons M.E., Heijenbrok-Kal M.H., CO-FLOW collaboration Group (2025). Health outcomes up to 3 years and post-exertional malaise in patients after hospitalization for COVID-19: A multicentre prospective cohort study (CO-FLOW). Lancet Reg. Health Eur..

[14] Berentschot J.C., Bek L.M., Heijenbrok-Kal M.H., van Bommel J., Ribbers G.M., Aerts J.G.J.V., Hellemons M.E., Berg-Emons H.J.G.v.D. (2024). Long-term health outcomes of COVID-19 in ICU- and non-ICU-treated patients up to 2 years after hospitalization: A longitudinal cohort study (CO-FLOW). J. Intensive Care.

[15] Lemhöfer C., Sturm C., Loudovici-Krug D., Guntenbrunner C., Bülow M., Reuken P., Quickert S., Best N. (2023). Quality of life and ability to work of patients with Post-COVID syndrome in relation to the number of existing symptoms and the duration since infection up to 12 months: A cross-sectional study. Qual Life Res..

[16] Barker-Davies R.M., Osullivan O., Senaratne K.P.P., Baker P., Cranley M., Dharm-Datta S., Ellis H., Goodall D., Gough M., Lewis S. (2020). The Stanford Hall consensus statement for post-COVID-19 rehabilitation. Br. J. Sports Med..

[17] Ceban F., Ling S., Lui L.M., Lee Y., Gill H., Teopiz K.M., Rodrigues N.B., Subramaniapillai M., Di Vincenzo J.D., Cao B. (2022). Fatigue and cognitive impairment in post-COVID-19 syndrome: A systematic review and meta-analysis. Brain Behav. Immun..

[18] Evans R.A., McAuley H.J.C., Harrison E.M., Shikotra A., Singapuri A., Sereno M., Elneima O., Docherty A.B., Lone N.I., Leavy O.C. (2021). Physical, cognitive, and mental health impacts of COVID-19 after hospitalisation (PHOSP-COVID): A UK multicentre, prospective cohort study. Lancet Respir. Med..

[19] Augustin M., Schommers P., Stecher M., Dewald F., Gieselmann L., Gruell H., Horn C., Vanshylla K., Di Cristanziano V., Osebold L. (2021). Post-COVID syndrome in non-hospitalised patients with COVID-19: A longitudinal prospective cohort study. Lancet Reg. Health Eur..

[20] Huang L., Yao Q., Gu X., Wang Q., Ren L., Wang Y., Hu P., Guo L., Liu M., Xu J. (2021). 1-year outcomes in hospital survivors with COVID-19: A longitudinal cohort study. Lancet.

[21] Eurofound (2020). Living, Working and COVID-19, COVID-19 Series. https://assets.eurofound.europa.eu/f/279033/5e54520687/ef20059en.pdf.

[22] Sykes D.L., Holdsworth L., Jawad N., Gunasekera P., Morice A.H., Crooks M.G. (2021). Post-COVID-19 symptom burden: What is long-COVID and how should we manage it?. Lung.

[23] Fatima S., Ismail M., Ejaz T., Shah Z., Fatima S., Shahzaib M., Jafri H.M. (2023). Association between long COVID and vaccination: A 12-month follow-up study in a low- to middle-income country. PLoS ONE.

[24] Nagin D.S. (2014). Group-based trajectory modeling: An overview. Ann. Nutr. Metab..

[25] Huang L., Li X., Gu X., Zhang H., Ren L., Guo L., Liu M., Wang Y., Cui D., Wang Y. (2022). Health outcomes in people 2 years after surviving hospitalisation with COVID-19: A longitudinal cohort study. Lancet Respir. Med..

[26] Garrigues E., Janvier P., Kherabi Y., Le Bot A., Hamon A., Gouze H., Doucet L., Berkani S., Oliosi E., Mallart E. (2020). Post-discharge persistent symptoms and health-related quality of life after hospitalization for COVID-19. J. Infect..

[27] EuroQol Group (1990). EuroQol—A new facility for the measurement of health-related quality of life. Health Policy.

[28] Tabachnick B.G., Fidell L.S. (2013). Using Multivariate Statistics.

[29] IBM Corp (2022). IBM SPSS Statistics for Windows, Version 29.0.

[30] EuroQol Research Foundation (2018). EQ-5D-3L User Guide. https://euroqol.org/publications/user-guides.

[31] World Health Organization WHO Global Health Observatory. https://www.who.int/data/gho.

[32] Ceravolo M.G., Anwar F., Andrenelli E., Udensi C., Qureshi J., Sivan M., Kiekens C., Zampolini M. (2023). Evidence-based position paper on physical and rehabilitation medicine professional practice for persons with COVID-19, including post COVID-19 condition: The European PRM position (UEMS PRM Section). Eur. J. Phys. Rehabil. Med..

[33] Donnelly J.P., Wang X.Q., Iwashyna T.J., Prescott H.C. (2021). Readmission and death after initial hospital discharge among patients with COVID-19 in a large multihospital system. JAMA.

[34] Lithuanian Ministry of Health COVID-19 Vaccination Strategy. https://sam.lrv.lt/lt/veiklos-sritys/visuomenes-sveikatos-prieziura/uzkreciamuju-ligu-valdymas/koronavirusas/covid-19-vakcinos-ir-vakcinacija/.

[35] World Health Organization (2022). Post COVID-19 Condition (Long COVID). https://www.who.int/europe/news-room/fact-sheets/item/post-covid-19-condition.

[36] Cuschieri S., Grech V. (2020). COVID-19 and diabetes: The why, the what and the how. J. Diabetes Complicat..

